# An integrated life cycle and water footprint assessment of nonfood crops based bioenergy production

**DOI:** 10.1038/s41598-021-83061-y

**Published:** 2021-02-16

**Authors:** Jun Li, Fengyin Xiong, Zhuo Chen

**Affiliations:** 1 School of International Relations, Sun Yat-Sen University, Guangzhou, China; 2 School of Management, Curtin University, Perth, Australia; 3 Key Laboratory of Energy Plants Resource and Utilization, Ministry of Agriculture, South China Agricultural University, Guangzhou, 510642 China; 4 State Key Laboratory of Urban and Regional Ecology, Research Center of Eco-Environmental Science, Chinese Academy of Sciences, Beijing, 100085 China; 5 Institute of Communication and Global Public Opinion, Xi’an International Studies University, Xi’an, 710061 China

**Keywords:** Climate-change ecology, Climate sciences, Energy science and technology, Engineering, Environmental impact, Risk factors, Climate-change impacts, Climate-change policy, Energy and society, Environmental impact, Sustainability

## Abstract

Biomass gasification, especially distribution to power generation, is considered as a promising way to tackle global energy and environmental challenges. However, previous researches on integrated analysis of the greenhouse gases (GHG) abatement potentials associated with biomass electrification are sparse and few have taken the freshwater utilization into account within a coherent framework, though both energy and water scarcity are lying in the central concerns in China’s environmental policy. This study employs a Life cycle assessment (LCA) model to analyse the actual performance combined with water footprint (WF) assessment methods. The inextricable trade-offs between three representative energy-producing technologies are explored based on three categories of non-food crops (maize, sorghum and hybrid *pennisetum*) cultivated in marginal arable land. WF results demonstrate that the Hybrid *pennisetum* system has the largest impact on the water resources whereas the other two technology options exhibit the characteristics of environmental sustainability. The large variances in contribution ratio between the four sub-processes in terms of total impacts are reflected by the LCA results. The Anaerobic Digestion process is found to be the main contributor whereas the Digestate management process is shown to be able to effectively mitigate the negative environmental impacts with an absolute share. Sensitivity analysis is implemented to detect the impacts of loss ratios variation, as silage mass and methane, on final results. The methane loss has the largest influence on the Hybrid pennisetum system, followed by the Maize system. Above all, the Sorghum system demonstrates the best performance amongst the considered assessment categories. Our study builds a pilot reference for further driving large-scale project of bioenergy production and conversion. The synergy of combined WF-LCA method allows us to conduct a comprehensive assessment and to provide insights into environmental and resource management.

## Introduction

Water scarcity, together with energy crisis and other environmental pressure such as air pollution has soared and affected the socioeconomic development of China^1,2^. Fossil fuel combustion related carbon emissions weigh a significant share in China's GHGs emissions, in which a majority part is attributed to the transport sector while still relying heavily on conventional fuels. Appropriate measures need to be implemented timely to optimize the nation’s energy supply structure to achieve the long term climate stabilization target^3^. In this regard, China has committed to increasing the share of non-fossil and reaching CO_2_ emissions peak by 2030 and carbon neutral economy by 2060, in compliance with the Paris Agreement^4^. Bioenergy, as a promising alternative, is expected to replace more than half of fossil energy^5^. And the global interest in bioenergy has grown to fill up energy gap^6^. Both China and the developed countries, the EU and the US among others, have committed to enhancing the share of bioenergy, such as biogas and biodiesel, through various policy incentives. For example, large-scaled biogas projects in China receive currently an average subsidy of 2500 CNY(360 US$) m^−3^ biogas, 0.25 kwh^−1^ electricity or 1500 CNY (215 US$) for each anaerobic digestion device^4^.

Biogas, with easier compression storage and higher security, is especially characterized by its superiority of energy conservation and emission reduction, has been vigorously promoted throughout different world regions^7,8^. However, the scientific community has reached a general consensus that biogas, mainly obtained from the anaerobic digestion of agricultural waste, organic waste and sewage^9^, has difficulties in feedstock supply (including biomass collection and distribution and instability of resources^10–12^. The relevant technology roadmaps, in regard to biogas system fed with organic feedstock, have been widely recommended^13^. The expansion of this measure is considered to contribute to ensure constant raw materials. Such non-food crops as sorghum, maize or energy grass are mostly preferred by means of abundant contained carbon to the benefit of biogas production^14–16^, together with higher storage capacity and availability^17,18^.

Currently, this kind of biogas production makes up 15% of biomass energy production in Germany which has set a target of 10 billion m^3^ in 2030^19^. Similar situation also happens in Poland where the share of biogas from agricultural waste has increased to 32% in total biomass supply by 2015^20^. However, an imminent concern has been raised as to whether biogas production will pose threats to the global food security^21^. Subsequently, a sustainable solution has been proposed by producing biomass feedstock on marginal land to relieve the conflict with traditional agriculture. Some forecasting studies suggest that the implementation of non-food biomass production in China would satisfy have a potential of over 290 million tonne of oil equivalent (mtone) each year with full utilization of marginal land^22^, which is equivalent to nearly 10% of total primary energy supply in China.

Currently, water resource stress and environmental degradation associated with energy production have received heightened focus where the interconnection among these three pillars should be further analysed for more harmonious coexistence^23,24^. Nevertheless, few studies in previous literature have addressed the water consumption issue in relation to the supply chain of bioenergy production based on an integrated approach. Therefore, an in-depth quantitative assessment is necessary to in order to improve the comprehensive management of biogas-related water consumption. In this regard, water resource flowing into the biogas production process should be rigorously investigated on a life cycle basis^25,26^. Water footprint (WF), an explicit multi-dimensional indicator measuring freshwater appropriation volumes by resource or pollution category, has been identified as a principal tool for water resources quantification analysis^27^. The notion of WF, initiated by Hoeksta, mainly reflects the total freshwater water consumptive volume incorporating direct and indirect water^28,29^. In the present study, WF is taken into consideration to determine water consumption within system boundary.

A majority of previous researches have either applied LCA, as a fundamental tool to clarify the relationship between biogas production and environmental performance^30–32^, or integrated with economic cost^33–35^ of bioenergy production. These literatures have revealed that the single method has some limitations and incompleteness. Meanwhile, the authors recognized the importance of examining the freshwater resource consumption associated with large-scale renewable energy supply. The integrated LCA and WF assessment methods, can therefore offer a promising way of reconciling water and energy studies within a comprehensive methodological framework^36^. This combined approach has been widely implemented in crops cultivation fields whereas its application in renewable energy production is rather scant^37–40^. Although WF calculation has been reflected in various studies of crop cultivation, few have applied it to the field of energy production and waste management studies. Furthermore, most of these researches mainly calculate the water consumption under an ideal condition^26^. Therefore, an improvement in WF calculation methods may avoid or minimize the degree of deviation in quantitative assessment.

To bridge the research gap identified in on the above discussion, this paper presents a case study based on the integrated LCA-WF methods to develop an improved integrated LCA approach for environmental sustainability assessment^41^. Some quantitative assessment of environmental performance and water source consumption have been carried out to screen out the “hotspots” through the whole biogas production process.

## Materials and methods

Three primary non-food crops, Maize, sorghum and *hybrid pennisetum* are being extensively cultivated our studied geographic region, i.e., Penglai City (37°N Lat, 120°E Long, Altitude 20 m, Rainfall 576.9 mm), located in the eastern part of China’s Shandong Province. These three representative crops belong to the representative crops of Starch, Carbohydrate and Cellulose class, respectively. Sorghum has been rapidly developed since it has been listed the non-grain energy crops approved by the government. This crop had the similar yield potential to maize, especially in drought resistance^42,43^. Hybrid *pennisetum*, as the hybrid product from *P. americahum and P. Purpureum*, member of *Pennisetum* family*,* is also strongly adaptable to the infertile marginal soil in the northern Chinese plain where the return to the planation of staple food and other cash crops (such as fruiters) is reportedly low. The typical feature of this crop is reflected by its high cellulose content of 36.15%, equivalent to the maize. C/N ratio reaches 27.54:1, close to the optimum anaerobic digestion ratio of (20–30:1) that was reported in previous research^44^. In addition, all of these three crops have efficient photosynthetic performance, which is particularly suitable for crop cultivation and management. This finding has also been tested and corroborated in previous experimental results under various conditions^45^. Therefore, these three crops are identified as the most promising crops among other energy crops.

The integrated LCA -WF method is employed in this paper. The main modelling structure proceeds as follows: (1) Defining the system boundary to clarify the specific input and output. (2) Adopting LCA for environment impacts analysis from various sub-process. (3) Implementing the water footprint assessment for water resource consumption quantification. (4) Conducting sensitive analysis to demonstrate the impacts of data parameter variation on final results. Furthermore, ReCiPe and IPCC method are applied to evaluate the environmental problems categorized with multiple impacts. Finally, we discuss some optimization issues arising in water footprint calculation in regards to how to take grey water footprint (WFgr) into consideration in an accurate manner, as pointed out by^26^.

### Goal and scope definition

**Figure 1:**
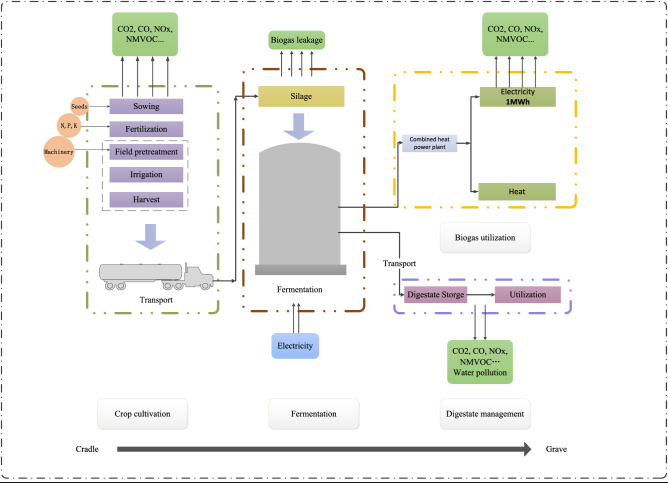
System boundary of three bioenergy production systems.

Since early 2000s, the Chinese National Energy Administration has launched a nationwide campaign to accelerate the combined heat and power (CHP) technology deployment for bioenergy conversion to realize the optimization of the country’s energy supply structure and to maximise the energy efficiency. This end-use utilization measure is characterized by certain practical significance with a high biogas utilization efficiency of 70–80%^46^. The functional unit (FU), as the basis of the system research, is expressed as 1MWh electricity corresponding to this motivation. The detailed system boundary of our study is described in Fig. 1. Furthermore, the involved distribution principle relies on economic value that is recommended in the ISO 14040, allowing the system analysis to reflect the share of environmental burden from relevant materials.

### Life cycle inventory

In the life cycle inventory part, the corresponding input and output flow chart are presented where the relevant details have been compiled within the system boundary^41^. The related data are collected from field investigation (the input of crop cultivation), the Ecoinvent database (background data) as well as the lab experiment data (with the major biogas and crop physicochemical characteristic).

#### Crop cultivation

The studied three non-food crops, i.e. Maize, Sorghum, and hybrid *pennisetum* are cultivated in the marginal land. To simplify our research hypothesis and data processing without loss of generality, it is assumed that carbon emission and uptake associated with land use change remain at an equilibrium and stability level.

The diesel consumption for ploughing, harrowing as well as the harvesting has been published in aggregate terms in local statistical yearbook and reported by field investigation in several Northern provinces in China. The pollutants emissions are calculated in line with emission coefficient whereas the machinery wastage of agricultural vehicles has been excluded in our analysis (which is very small compared with the direct emissions from the major processes of crops production). Additionally, chemical nutrients and pesticide during the cultivation process, being the non-negligible input for growth and yields, are systematically considered in the LCA analysis. The determination of the actual consumption relies on the absolute nutrient balance, depending primarily on the soil and crops characteristics. These materials expansion is accompanied with the nutrients loss and pollution transfer. Assumptions are being made to take the nitrogen content of fertilizers as reference. The corresponding pollution values are then obtained from the fertilizer efficiency equation.

As far as the water usage for irrigation is concerned, the deep groundwater is pumped at a distance to meet the crops demands. For the sake of brevity and simplicity, 1 m^3^ of water pumped from a depth of 500 m is assumed while the pumping efficiency is set as 80% with an average electricity consumption of 1.7kWh based on the work and energy equation^47^. In addition, the plastic film pollution is disregarded as a result of low utilization and lack of data sources. The demand for the seeds depends on the crop requirement and seed quantity. For illustrative purposes, it is assumed that the seeds are supplied from natural sources rather than organic production without upstream inputs consideration. A default distance of 20 km is set for transporting the crop and digestate to the production destination. The determination of destination pertains to relevant scenario simulation, where the distance from field to biogas plants and to the digestate management sites has been both hypothesized as 20 km. This assumption has been built on the scale required for the development of industrialised Biogas generating plants which integrate the feedstock cultivation with the downstream digestate management.

#### Anaerobic digestion

This sub-process scope derives from the crops silage to biogas production. After transported to the destination, the crops are stored for an extended period mainly optimization to the top state where the total weight is assumed to decrease 10% regardless of trace pollution. Anaerobic digestion (AD) is implemented under mesophilic conditions of 37 °C with the premise of suitability and lower energy consumption. The proportion between the volatile solid (VS) of materials and inoculum is chosen as 2:1. The inoculums, produced as part of the waste materials with high-degree moisture, are assumed to have neutral impacts on the environment in the current study. The crop characteristics of the anaerobic digestion are measured and shown in the Table 1 on the basis of external experimental results. Besides, the details on specific energy consumption to preserve normal external machine operation are provided in Online Appendix A1.

**Table 1:** Summary of crop characteristics.

Crop	DM content (t)	CH4 content (%)	Biogas yield (m^3^ t^−1^ TS)	TS (% mass)
Maize	33.5	60	196.7	0.223
Sorghum	30.5	50	334.58	0.442
Hybrid pennisetum	33	63	187.53	0.22

DM: dry materials, the measured weight of materials, removal of free water, is kept constant after drying.

TS: total solid, organic and inorganic matters embodied in the materials.

VS: volatile solid, organic matters embodied in the raw materials.

#### Digestate management

The gas-tight tank is considered for digestate storage in some references resorting to minimizing these residual emissions. However, many biogas plants remain to collect the anaerobic digestion residue (digestate) in open lagoons or tanks, though ammonia methane and nitrous oxides are emitted as the digestion process continues in open containers. This method of storing digestate in open tank is preferred as it is in line with physical property for biogas plants. Besides, it has been widely debated whether the residue emissions will be eliminated or just temporarily accumulated in gas-tight tanks. Therefore, an open tank storing the digestate for a period of 150–180 days, would generate such GHG emissions as methane, ammonia and nitrous oxide. The processed digestate, as a promising alternative to substitute the chemical fertilizers, has been modelled within an enlarged system boundary in Simapro, and will be consequently transported to a designated destination for further application. The relevant data of substituting fertilizers has been presented in Online Appendix A3, concerning Phosphate, nitrogen and potassium fertilizers, where the corresponding emissions associated with the digestate utilization have also been reported. The specific environmental credits are further determined by subsequent allocation and physicochemical property where the allocated environmental benefits subtract the avoided pollutants caused by the substitution of fertilizers for the final assessment of environmental impacts. The allocation is a critical issue as the biogas is deemed as the main by-product accounting for 80%, 20% of digestate, referring to the economic values embodied into these two materials^48^.

#### Biogas utilization

The biogas is supposed to supply the internal combustion for generating electricity combined with heat. Currently, the energy efficiency of biogas internal-combustion generator may reach 32%. This conversion efficiency ratio is utilized to measure the various biogas volume due to the non-identical methane content. The biogas is supposed to be purified and then injected into the gas network, where the CO_2_, PPM, H_2_S, water and minute N_2_.H_2_S will be removed by means of various deployments in the regeneration tank. The relevant emissions associated with biogas purification and compression are provided in Online Appendix Table A4, where the pollutants were mainly calculated on the basis of considered factors relating to purification and upgrading processes with reference to reviewed literature. The biogas leakage effect is excluded from our analysis and are not incorporated into the life cycle inventory of its micro-pollution characteristics^49,50^.

### Water footprint

The Cropwat software, developed by the Food and Agriculture Organization (FAO), has been widely adopted in numerous WF researches^10,28^. However, the results associate with WF_b_ were calculated under the ideal state, neglecting the wastage during the irrigation period^51^. Therefore, the combined method of integrated Cropwat and formula is applied for analysing the practical water flows.

The WF_g_ consumption is dependent on the rainfall, generated by evaporation stored in the soil or the surface of vegetation temporarily for crops growth^52^. In other words, WFg from anaerobic digestion and biogas utilization processes were not considered in the WF calculation. The data of daily rainfall, relative humidity, average wind speed, maximum and minimum temperature is collected through meteorological stations report for WFg estimation, which is then calculated by Eq. (1)^26,53^:

1 $${\text{WF}}_{g} = \frac{CWU}{Y} = 10 \times \frac{{min\left( {ET_{c} ,P_{eff} } \right)}}{Y}$$

where *ET*_*C*_ represents the potential evapotranspiration during reproductive periods (mm) while *P*_*eff*_, *Y* are defined as valid rainfall (mm), fresh weight (kg/hm^2^) respectively, and 10 is taken as the unit conversion coefficient. Equation (2) from the American soil Conservation Service is employed for the effective rainfall determination where *P* is the reproductive rainfall (mm).

2 $$P_{eff} = \left\{ {\begin{array}{*{20}c} {\frac{{P\left( {41.67 - 0.2P} \right)}}{41.67} P \le 83.33 } \\ {41.67 + 0.1P P > 83.33 } \\ \end{array} } \right.$$

3 $$ET_{c} = ET_{0} \times K_{c} \times K_{s}$$

The newly added *K*_*s*_ is included to depict the soil moisture condition, reflecting the symptom of water supply diversity wherein the values embedded into the software are quoted. Other parameters of both *ET*_*0*_ and *K*_*c*_ were referred to previous literature^54,55^. The *K*_*c*_ represents the crop coefficient, reflecting the influence of crop’s physiological shape and cultivation conditions on the water demand, which is relatively constant. The meaning of *ET*_*0*_ (mm/day) is introduced to express the daily evapotranspiration of crops, calculated by Cropwat software with the main parameters of temperature, sunshine hours, weed speed, humidity.

The impacts of following external factors on crops have been considered for WF_b_ calculation, as shown in Eqs. (4)–(6).

4 $$WF_{b} = \frac{{CWU_{b} }}{Y} = 10 \times \frac{{ET_{b} + L}}{Y}$$

5 $$ET_{b} = max\left( {0,ET_{c} - P_{e} } \right)$$

6 $${\text{L}} = \frac{{\alpha \times ET_{b} \left( {1 - \mu } \right)}}{\mu }$$

*ET*_*b*_ (mm) is the considered water evapotranspiration to address the blue water of field crops. L (mm) refers to the consumptive loss of the irrigation water during the transmission process and relates to the amount of irrigation water and the soil condition. Its estimation is drawn on Eq. (6) to account for the complex performance of irrigation water consumption. $\mu$ is utilization coefficient of irrigation water to weight the effective utilization of irrigation water which is derived from the actual measurement and equals to 0.73^56^. Moreover, taking the value of 5%, implies the proportional loss of water surface evaporation in water delivery process^57^.

WFgr represents the water pollution associated with fertilizers utilization through the crop cultivation process. Based on the standards from EPA, less than 10 mg nitrogen per litre should be employed^58^. The impacts of pesticide on water quality have not been taken into consideration due to lack of relevant data. Equation (7) has been employed to calculate this parameter:

7 $$WF_{{{\text{gr}}}} = \frac{{\left( {\alpha \times AR} \right)/\left( {Cmax - Cnat} \right)}}{Y}$$

${\upalpha }$, reflecting the refraction purity from nitrogen fertilizer, is set as 25%. C*nat* acts as natural background concentration of pollutant while *Cmax* as the maximum concentration allowed in the environment, assumed to be 10 mg/L and 0 respectively. Besides, AR (kg/hm^2^), as the fertilizer applied to each hectare, is set as the basis of WFgr calculation^59^.

Furthermore, this paper exploits the general phase accumulation method to calculate the industrial water footprint by considering both the direct inputs and indirect water consumption. In principle, avoided water footprint should be taken into account likewise in the life cycle assessment methods. However, it was advised by some literatures to neglect the avoided contribution mainly due to lack of relevant data^26,60^. Consequently, it remains an important research gap which is worthy being further explored. Some supplement is raised that the water footprint also demands to be distributed based on the economic value, similar to LCA.

Generally, the labour related water footprint is neglected in our WF analysis framework. It would cause the double or inflate the WF calculation leading to inaccuracy if considered^57^. The transportation-related water footprint is not employed either to given its negligible demand for water resource^61^.

## Results and discussion

### Life cycle assessment

The ReCiPe Midpoint method is applied for environmental impacts assessment. The positive values denote environmental burden while the negative values represent environmental credits from LCA critique perspective.

#### Detailed assessment results in ReCiPe method

**Figure 2:**
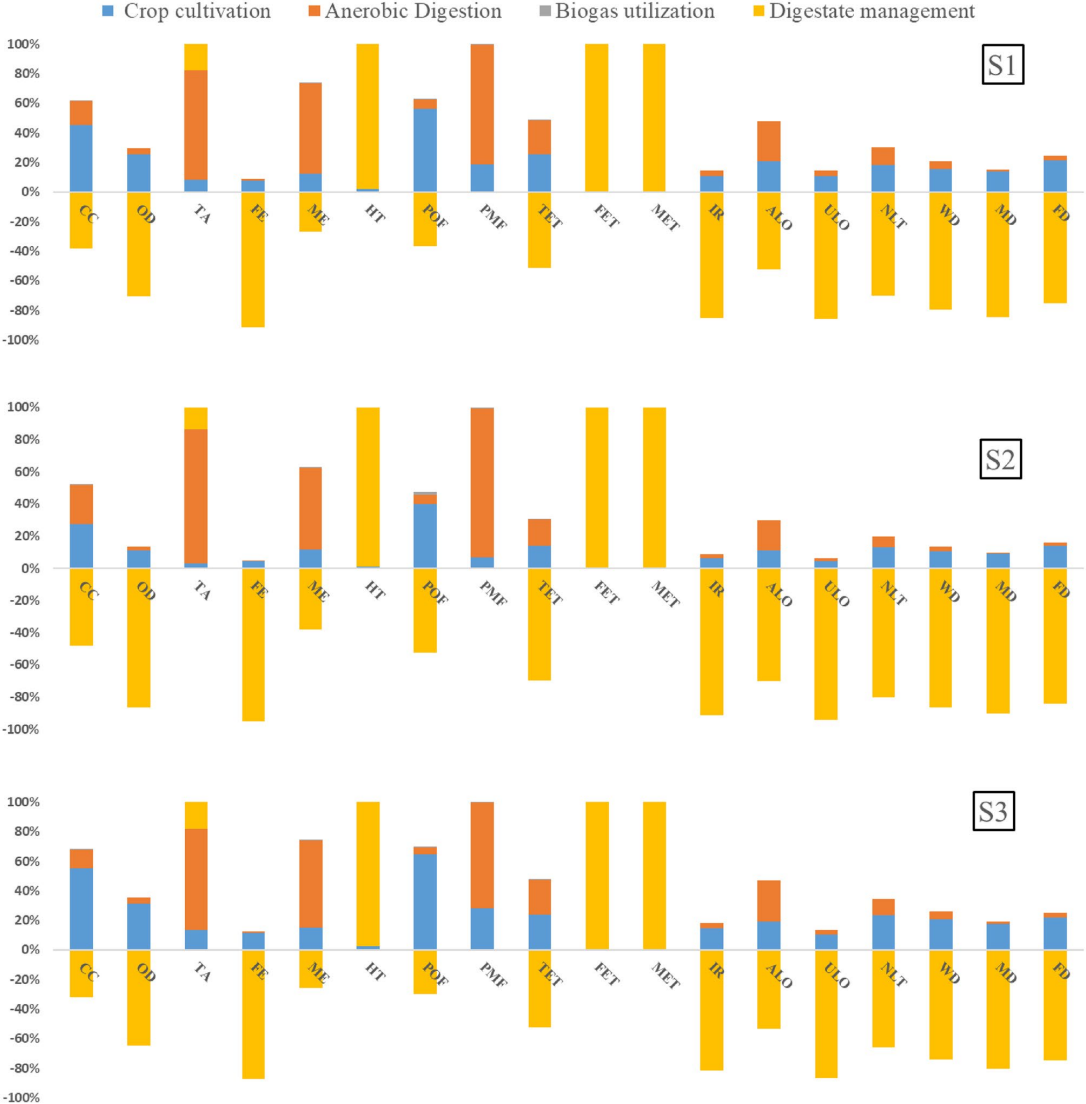
Characterization results from four various sub-process of each system.

The characterized results are illustrated in Fig. 2 which reveals the varied contributions of four sub-processes to each impact category. The sub-process that has larger contributions to the overall environmental impacts is preferentially elaborated thanks to its better representativeness.

The actual environmental burdens in cultivation presented by maize system (S1) mainly concentrates on CC, OD, POF, TET within the range of 25–56% where a large number of materials inputs relating to agricultural behaviour generate these impacts. More attention is deserved to be paid for subsequent improvement in agricultural pattern. Relative major environmental impacts are reflected by sorghum system (S2) on CC (28%) and POF (40%). The raw materials required lowest from per functional unit among these three systems, that is, performs relatively minimal environmental impacts. By contrast, hybrid Pennisetum system (S3) causes significant total environmental impacts, ranging from 18 to 64% covering 8 categories, especially PMF being a remarkable hotspot. Specifically, irrigation farming activities of this system shows distinct influences on NLT and WD resulting from the highest blue water footprint. Other agricultural practices, such as fertilizer or machinery utilization for field preparation and harvest, are closely interrelated to the rest of impact categories by means of long growth cycle. Above all, the assessment results suggest that hybrid *pennisetum* system have major impacts on the environment from single analysis perspective.

AD results of S1 illustrate a more serious influence on certain kinds of categories, such as TA, ME, PMF with the specific values of 74%, 61%, 81% respectively. The primary reason relates to the ME indicator where the nitrogen emissions, i.e., the whole life cycle of NOx and NH3, are identified as the calculation reference. Mesophilic condition is maintained for smooth operation in anaerobic digestion process where diesel and electricity are utilized for achievement. Digestate storage are also incorporated for consideration where organic materials’ chemical reaction exists to generate emissions pollution. Furthermore, these actual situations also aggravate PMF and TA potentials. The environmental burden of S2 system is mainly reflected by resulting TA, ME and PMF which range between 51 and 93%, and fossil energy consumption appears to account for a large amount of fugitive pollution emission. On the contrary, S3 exhibits minimum influence to the total assessment, the contribution ratio to the TA, ME, PMF with the range of 59–72%. Overall, these assessment results reveal a remarkable detrimental impact to groundwater, causing serious acidification and eutrophication among three systems. In comparison, the result from S3 generated far less damage as compared with the aforementioned two systems.

In terms of digestate management process, the absolute share of the environmental burden and benefits among these three systems have been presented. The negative values imply the ability of mitigating the environmental pollution resulting from mineral chemicals generation. The main share of avoided environmental pollution categories varieties is found to be attributed to S3 system, subject to the maximum digestate production. Nevertheless, some severe environmental effects such as HT, FET and MET with the absolute values are also proposed to be explicitly addressed. Such extreme adverse impacts on the groundwater and human health are caused by large quantities of metal ions transported through leaching. Similar trends appear to other two systems with an analogous rationale. Therefore, a new usage plan is encouraged to be developed for improved environmental management.

Figure 2 also suggests that both the positive or negative environment impacts from biogas utilization are much less than the other three processes. Note that all environment impacts generated by biogas utilization process did not take explicit consideration of the heat recovery to keep our analysis framework succinct and transparent. Once heat recovery consideration is involved in the LCA process, the related energy, materials consumption and recycling efficiency should be appropriately taken into account. However, the data limitation and unavailable recycling energy information would increase the uncertainty and inaccuracy in final assessments, rendering the results more difficult to interpret.

#### Comparative analysis with IPCC method

**Figure 3:**
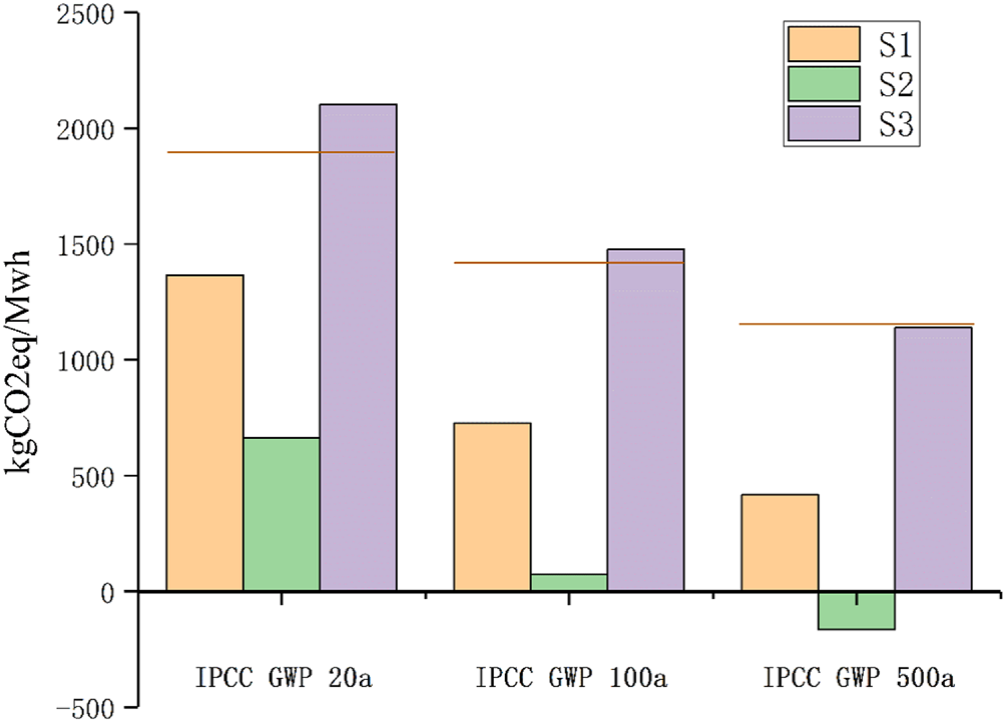
GHG impacts in various time horizon of each system based on IPCC methods in comparison with market electricity under the same functional unit conditions.

It is highlighted that bioenergy produced from the energy crops need to reduce the GHG emission relative to fossil fuels by 70%, a reference value recommended by the European Commission’s bioenergy development guideline^62^. Some noteworthy suggestion is presented that fossil fuels, as the reference substance likewise the report suggestion, are not adopted because of system boundary different to this research. The total carbon footprint among these three systems and electricity generation were, therefore, calculated for comparison purposes on account of IPCC 2013 methods. More specifically, three evaluation methods with different time horizon have also been applied with representative carbon dioxide equivalent factors, where the actual impacts of the systems on climate change are shown in Fig. 3.

There is a major finding to be addressed that three systems do not have worse performance than electric power grid assessed by IPCC 20a (20 years) assessments results in absolute terms. Detailed examination suggests that S1 and S2 would achieve a better result of GHG emission reduction, S2, in particular, appeared to nearly triple the emissions reduction relative to the reference system in numerical values. Therefore, the above two systems could be recommended for sustainable environmental development and GHG reduction. However, S3 generates a relatively significant degree of GHG emission, slightly higher than the proposed reference. GHG emission is found to be closely correlated to each sub-process, especially AD process with a decisive role in accordance with abovementioned characterization results analysis. Along with the pollutants discharge in digestate storage that is also incorporated into consideration, of high influence to final results. The environmental burden in AD process, therefore, had shown up compared with the rest. These three systems all appears on a various levels of drop state. S1 and S2 results perform far below the reference system of GWP 20a and GWP 100a assessment. The GWP from S1 is decreased by 69.4% from 20 to 500a horizon while 46% reduction is observed for S3. Besides, the negative numerical values after subtracting system carbon dioxide equivalence, that is, the extrapolated S2 can be considered as carbon sink to some degrees. Note the estimation of emissions are expressed in carbon equivalent terms.

The above assessments provide some important but not exclusive indicators. Subsequently, the details of a comprehensive comparison across all impact categories are presented in Table 2. With regard to TA, an indicator that reflects the changes in chemical properties of soil and resultant damages to the ecosystem, close relevant to the nitrogen-based or sulphur dioxide emission. These three systems are characterized with similar contribution except for S3 in which a slightly worse impact is embodied. Specifically, the central focus mainly concentrates on the first two sub-processes, i.e. crop cultivation and Anaerobic Digestion. The calculation results indicate that the revealed numerical values from AD process are much larger than crops cultivation process, accounting for an absolute proportion. Besides, the cultivated feedstock *f*, in which abundant carbon is embodied, is observed to be the main driver for biogas potential exploitation. Accordingly, uptake and leaching were chiefly driven by fertilizers applications which naturally enhance environmental burden. Consequently, S2 exhibits the best performance amongst the three considered systems from this perspective.

**Table 2:** The characterization results between these three systems and market.

Impact category	Unit	S1	S2	S3	Market
CC	kg CO2 eq	715.54	50.93	1470.58	1396.53
OD	kg CFC-11 eq	0.00	0.00	0.00	0.00
TA	kg SO2 eq	143.57	111.12	155.75	7.15
FE	kg P eq	− 1.01	− 0.62	− 1.07	0.20
ME	kg N eq	43.02	9.39	51.70	0.18
HT	kg 1,4-DB eq	7724.71	4487.60	8666.75	199.82
POF	kg NMVOC	3.72	− 0.29	7.87	3.96
PMF	kg PM10 eq	17.28	13.10	19.60	3.05
TET	kg 1,4-DB eq	− 0.03	− 0.18	− 0.05	0.02
FET	kg 1,4-DB eq	170,664.69	99,994.72	190,162.99	5.23
MET	kg 1,4-DB eq	146,056.04	85,576.04	162,743.04	5.12
IR	kBq U235 eq	− 172.41	− 110.41	− 179.63	2.21
ALO	m2a	− 9.15	− 35.30	− 13.69	19.36
ULO	m2a	− 56.79	− 37.32	− 64.01	12.26
NLT	m2	− 0.13	− 0.10	− 0.12	0.05
WD	m3	− 17.03	− 11.40	− 16.68	1.96
MD	kg Fe eq	− 166.31	− 105.80	− 171.34	4.16
FD	kg oil eq	− 277.80	− 196.59	− 304.46	276.45

It is advised in previous literature that HT may cause potential harms through its accumulation and persistence in the environment, and its chain effect may further damage the ecosystem diversity and human health. The higher inputs of chemical substance and energy consumption, the more severe negative impacts the chemical process may result in. In this context, digestate management process is thus highlighted as a hotspot for further analysis with its significant share in environmental health degradation. A large amount of heavy metal ions remains soil or leaches to groundwater, causing long-term damages to human health. Therefore, strong efforts need to be mobilised for a better digestate management to alleviate negative environmental impacts. From the perspective of PMF, these three systems are characterized with a slightly worse performance than reference especially S3, weighted by such emitting substance to environment as GHGs and primary particulate. The interpretation of ALO is points to the agricultural land area occupied with a certain length of time. The numerical values are all negative, suggesting beneficial impacts as compared with the reference.

### Water footprint analysis

The WF calculation is composed of three parts and the respective results are formulated by specific algorithm. These three- category WF results are reported in Table 3.

**Table 3:** WF results from three systems.

Crop	Process	WF_g_	WF_b_	WF_gr_
Maize	Crop cultivation (m^3^/kg)	0.043	0.052	0.105
	Anaerobic digestion (kg/m^3^)		0.42	1.2
	Biogas utilization (kg)		− 2.89	− 6.43
Sorghum	Crop cultivation (m^3^/kg)	0.052	0.035	0.119
	Anaerobic digestion (kg/m^3^)		0.12	0.34
	Biogas utilization (kg)		− 2.83	− 6.3
Hybrid pennisetum	Crop cultivation (m^3^/kg)	0.04	0.11	0.16
	Anaerobic digestion (kg/m^3^)		0.49	1.42
	Biogas utilization (kg)		− 3.17	− 7.05

Table 3 indicates that WF_g_, which is mainly reflected in crops cultivation, only exhibits non-significant difference among these three systems. The sorghum consumes much more water contained in the soil than other crops, consistent with the results from Water stress index in life cycle assessment. Maize are characterized with relatively lower WF_g_ in comparison with the same crops cultivated in common soil resulting from higher yields. From a single point perspective of WF, this system is equipped with a certain sustainability^63^. WF_b_ results show that S3, with a strong presence, consumes most of groundwater or surface water mainly in anaerobic digestion phase. WF_gr_ assessment, similar to WF_b_ in order, is bounded by some limitations such that this calculation only takes the nitrogen volume application as a reference. In short, S2 turns out to exhibit the optimal performance in terms of water resource utilization. However, it is deserved to point out that some uncertainties still constrain the WF calculation. The proper allocation between the biogas and digestate should be determined following the economic or energy efficiency principle as well as life cycle assessment theory.

### Sensitivity analysis

The important data parameters should be altered to observe the variety slope on final evaluation results under the change of same magnitude. Multiple parameters variation are then applied to the three systems to test the sensitivity of model’s input data in terms of environmental impacts.

#### Silage mass loss

**Figure 4:**
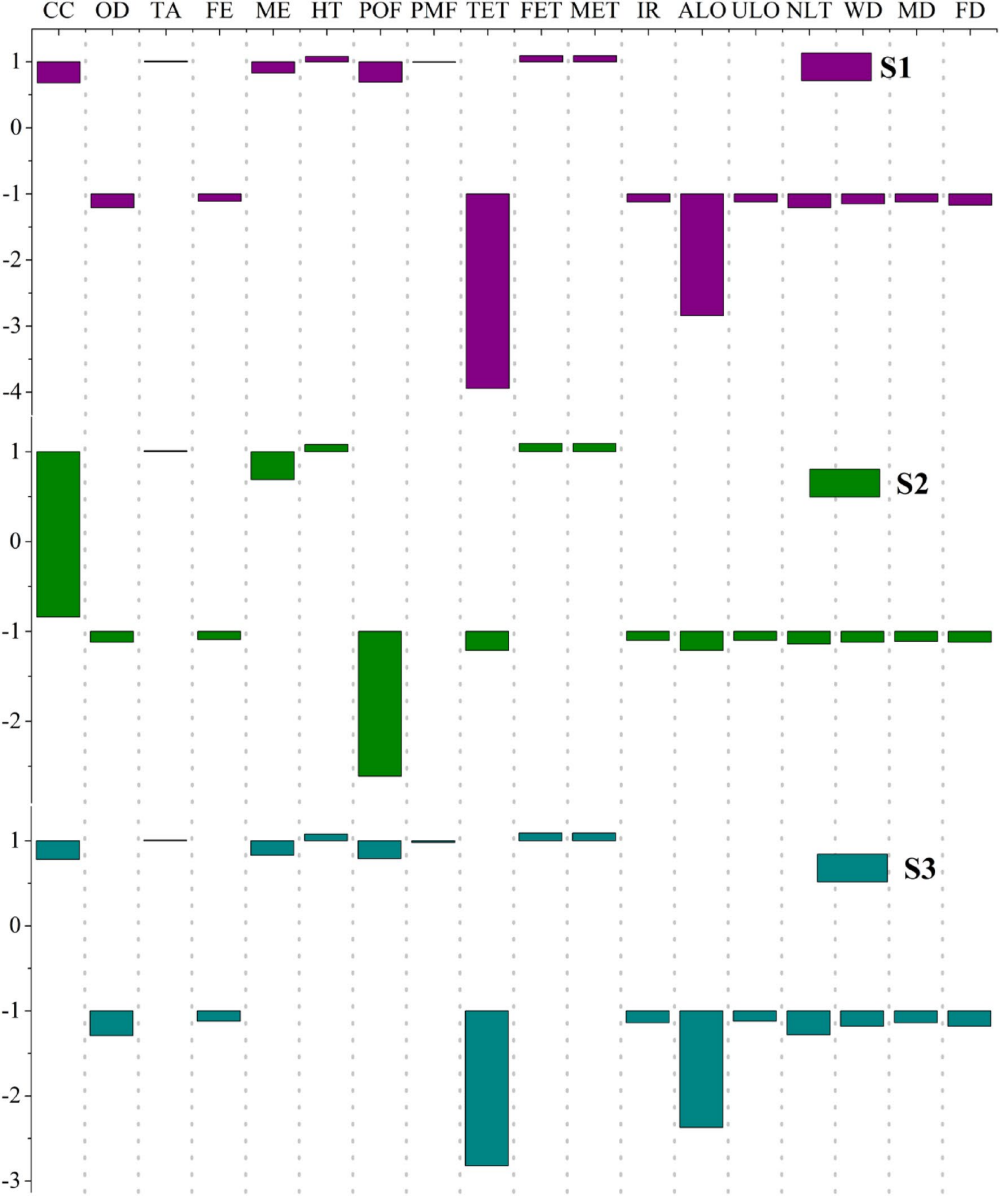
The sensitivity results of each system under various scenarios in ReCiPe assessment method.

The typical variable parameters pertaining to crops input quantity mainly occur during the silage process which may result in reduced crops production to a certain degree. Intuitively, the more raw material’s quantity loss exists, the more corresponding crops need to be cultivated in the light of mass balance. Consequently, the impacts of variations in critical parameters on the final environments impacts need to be carefully examined. The typical mass loss ratio in similar study framework lies within the range between 2 and 10%, reported by^64^. The 10% variation in silage loss is identified as the Baseline scenario whereas the ratio for the other two alternative scenarios was assumed to be 5% (SB) and 2% (SA), respectively. The results under various simulated scenarios are presented in Fig. 4**.**

Obvious drop appears in the CC impact categories in S1 with the corresponding value of 32% and 20%, comparing with the Baseline scenario results with 100% contribution as the reference threshold. Additionally, other categories with the positive influence are similarly strengthened, decreasing to 13%, 21% for OD impact categories on the original basis, and 11%, 17% for FD. Likewise, the relative sensitivity of impacts to the variation in S2’s parameters is also examined for the aforementioned categories. The corresponding decline, involving CC and impact category, is far more significant than the other two systems with 119%, 184%, respectively. Furthermore, S2 exhibits an obvious sensitivity to POF by alleviating the negative effects to a higher degree, unlike the other two systems whose performance generated serious adverse impacts. Slight variation is exhibited in multiple categories as TET, ALO in S2 whereas the same types of sensitivities are two or even three times larger in S1 and S3. It is noteworthy that the variation trend of PMF, TA, HT, FET and MET manifests strong consistency among these three systems, characterized with very subtle differences. Furthermore, environmental impacts in terms of HT, FET, and MET in various simulated scenarios have aggravated with corresponding amplification from each of them.

**Figure 5:**
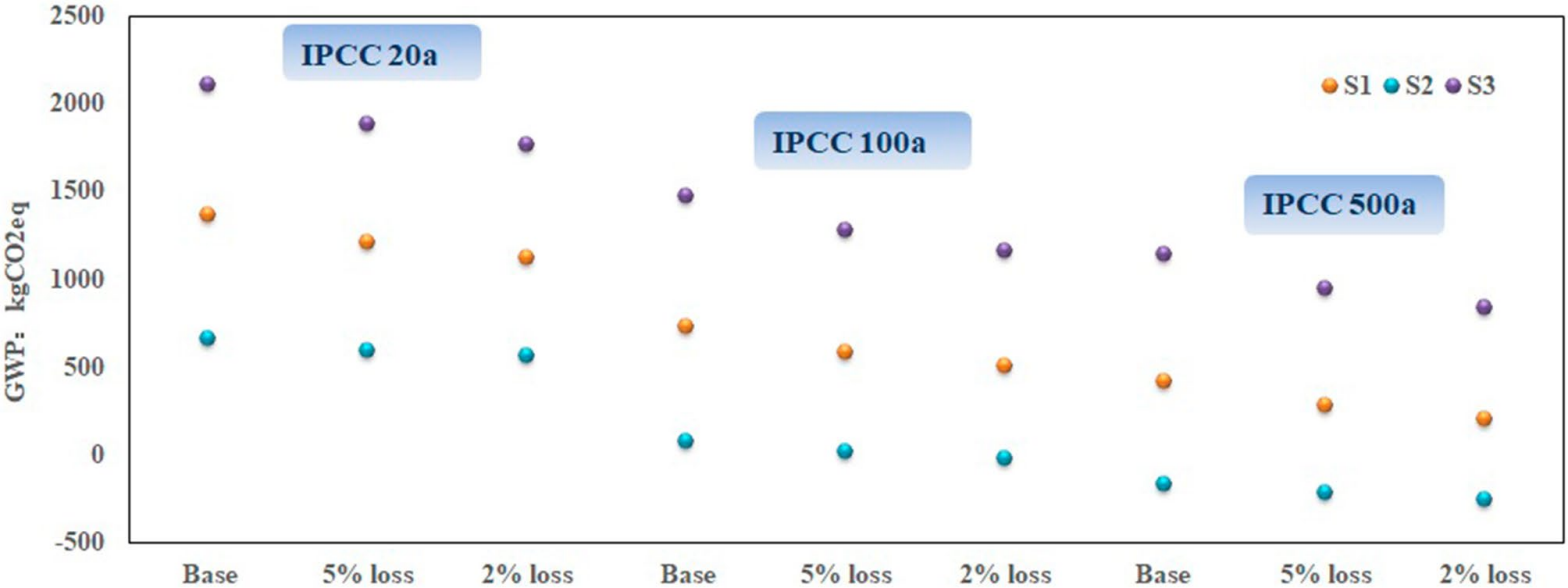
The sensitivity results of each system in IPCC assessment method under different situations of change in silage mass loss ratio.

The sensitivity trend consisting of the IPCC results under various time horizons is depicted in Fig. 5. The sensitivity of impact factors to the results is elaborated by the linear fitting with corresponding slope. The deeper the slope is, the more sensitive to tested parameters the results are, resulting from a certain linear interrelation existing between sensitivity and impact factors^65^. Each system responds to the three assumed scenarios with high consistency where most sensitive scenario appears in S3 followed by S1. Importantly, the underlying regularity pattern reveals that the sensitivity from silage mass loss factors would weaken over time from the long-term perspective.

#### Methane loss

**Figure 6:**
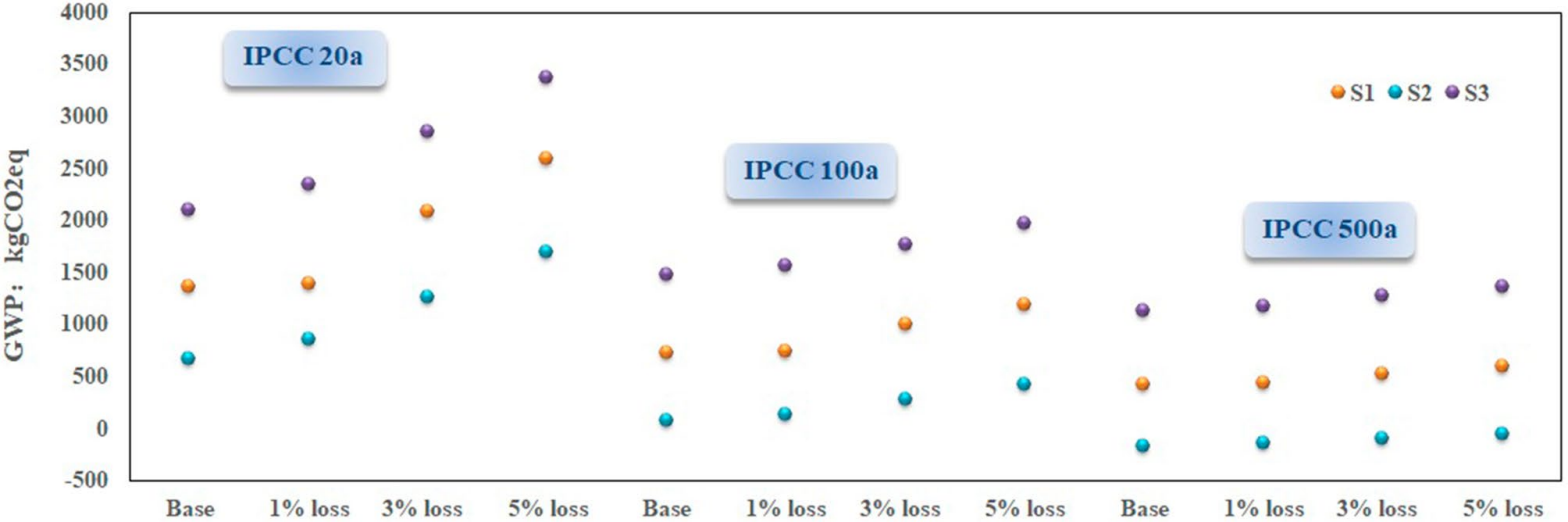
The sensitivity results of each system with IPCC assessment method under the situation of methane loss ratio change.

The methane leakage from biogas manufacturing supply chain not only contributes to increasing the GHG emissions to atmosphere but also affects the overall efficiency of electricity generation system. Typical methane escape ratio in chemical industry process is reported to lie within the range from 1.7 to 5.2% under the normal conditions^66^. In baseline scenario, no methane leakage is assumed where the biogas is fully delivered to the target generator set. Three other alternative scenarios are taken into consideration with specific leakage ratio of 1%, 3%, 5%, respectively. The IPCC methods from various time horizons are adopted likewise as in the aforementioned sensitive analysis. Accordingly, the resultant trends are outlined in Fig. 6, indicating an approximately linear relationship among the experimental points with the correction determination factor. To summarise, methane loss has predominant impacts on the final results of S3, followed by S1 based on the IPCC 100a 500a assessment methodology, which is consistent with the above sensitivity analysis in an orderly fashion. However, S1 appears to be the most sensitive scenario compared with the other two systems for the IPCC 20a assessment method.

Interestingly, such a sequence pattern with decreasing sensitivity with time horizon has been observed whichever specific assumed scenarios are embodied. Both decline and matching curve’s slopes in methane loss scenario significantly exceeds that of silage mass scenario from actual GWP 20a and GWP 100a results except for the GWP 500a case. In other words, the effect of leakage ratio changes is larger than the silage mass loss alteration on environmental impacts in the foreseeable future. These sensitivity test results can provide useful reference and guideline for decision making in bioenergy development by means of examining whether a specially selected scenario performs in accordance with environmental friendly criteria.

## Conclusions

The energy-water-environment nexus assessment, involving three energy crops-based (Maize, Sorghum, Hybrid Pennisetum) biogas conversion have been implemented in this study. LCA and WF methods are integrated for evaluating the actual performance in terms of environmental impacts and water resource consumption of biogas production systems based on three distinct non-food energy crops.

The LCA results, utilizing ReCiPe method, indicate that not every system has negative impacts in evaluated categories, and some systems have simultaneous positive effects. The environmental credits are mainly embodied in the digestate management part along with certain negative impacts such as FET, MET and HT. Therefore, some improvement and modification should be devoted to alleviating severity in this sub-process. IPCC assessment results demonstrates that S1 and S2, especially S2, are less than the local electricity generation while S3 is close to or just below the reference with the time horizon expansion. Therefore, these three systems are characterized with certain potentials in mitigating the GWP in the long term. Furthermore, GWP comparison among multiple scenario simulations from sensitivity analysis suggest that changes in methane leakage ratio have larger environmental impacts than the silage mass loss alteration.

WF results demonstrate relatively lower WF_g_ and WF_b_ in each crop cultivation process, comparing with the same crops grown in other types of soil. The sub-process contribution among these three systems demonstrate high consistency with the final results where the anaerobic digestion and crop cultivation process requires more attention to management owing to serious water resource depletion. In summary, S2 presents the best performance in water utilization and environmental pollution mitigation while S3 requires optimizing its water resource flow.

Some limitations have to be pointed out for further investigation, such that the regional disparity has not been considered in the present study, as it is known that the water allocation or management in different geographical regions may vary to some degrees. Nevertheless, the vast territory of China makes this hypothetical implementation on a spatial scale across the whole country unrealistic, as argued in previous literature on water footprint assessment^66^, Hybrid *Pennisetum* is not acclimatized to growth in some areas. The combined methods developed in this study and implementation primarily rely on the theoretical similarity and synergy. Some degree of uncertainty may arise in the simulated scenarios as a result of data commonality. In this regard, prudent adjustments and improvements based on reconciliation of these two assessment methods are required in the future studies.

To summarise, our modelling study indicates that Hybrid *Pennisetum* system has the most severe environmental impacts from an integrated assessment perspective. Besides, large amounts of labour have been allocated to energy crops cultivation in China, driven by the government’s lavish renewable energy subsidy scheme. However, economic rationale for energy crop should obviously be taken into account for large-scale cultivation of this crop in farmland. The other two studied systems manifested varied responses to the relevant impact categories, either negative or positive depending on the specific indicators and criteria, where of the appropriateness needs to be interpreted with caution.

## Supplementary Information

Supplementary Information

## Abbreviations

AD: Anaerobic digestion
ALO: Agricultural land occupation
CC: Climate change
CHP: Combined heat and power
DM: Dry materials
FAO: Food and Agriculture Organization
FD: Fossil depletion
FE: Freshwater eutrophication
FET: Freshwater ecotoxicity
FU: Functional unit
GHG: Greenhouse gas
GWP: Global warming potential
HT: Human toxicity
IPCC: Intergovernmental Panel on Climate Change
IR: Ionising radiation
LCA: Life cycle assessment
MD: Metal depletion
ME: Marine eutrophication
MET: Marine ecotoxicity
NLT: Natural land transformation
OD: Ozone depletion
PMF: Particulate matter formation
POF: Photochemical oxidant formation
TA: Terrestrial acidification
TET: Terrestrial ecotoxicity
TS: Total solid
ULO: Urban land occupation
VS: Volatile solid
WD: Water depletion
WF: Water footprint
WFb: Blue water footprint
WFg: Green water footprint
WFgr: Grey water footprint
