# Publisher Correction: An integrated life cycle and water footprint assessment of nonfood crops based bioenergy production

**DOI:** 10.1038/s41598-021-90390-5

**Published:** 2021-05-20

**Authors:** Jun Li, Fengyin Xiong, Zhuo Chen

**Affiliations:** 1 School of International Relations, Sun Yat-Sen University, Guangzhou, China; 2 School of Management, Curtin University, Perth, Australia; 3 Key Laboratory of Energy Plants Resource and Utilization, Ministry of Agriculture, South China Agricultural University, Guangzhou, 510642 China; 4 State Key Laboratory of Urban and Regional Ecology, Research Center of Eco‑Environmental Science, Chinese Academy of Sciences, Beijing, 100085 China; 5 Institute of Communication and Global Public Opinion, Xi’an International Studies University, Xi’an, 710061 China

Correction to: *Scientific Reports* https://doi.org/10.1038/s41598-021-83061-y, Published online 16 February 2021

In the original version of this Article, Zhuo Chen was incorrectly affiliated with 'School of International Relations, Sun Yat-Sen University, Guangzhou, China'. The correct affiliation is listed below.

Institute of Communication and Global Public Opinion, Xi'an International Studies University, Xi’an, 710,061, China.

