# Supplementary material for: An integrated life cycle and water footprint assessment of nonfood crops based bioenergy production
Source: Sci Rep. 2021 Feb 16;11:3912. doi: 10.1038/s41598-021-83061-y (PMC7887239; doi:10.1038/s41598-021-83061-y)
Supplement: Supplementary file 1 — Supplementary Information [file 41598_2021_83061_MOESM1_ESM.docx]

**An integrated life cycle and water footprint assessment of nonfood crops based bioenergy production**

Jun Li ^a, e *#^,Fengyin Xiong ^b,d#^  Zhuo Chen ^c *^

^a^ School of International Relations, Sun Yat-sen University, Guangzhou, China.

^b^ Key Laboratory of Energy Plants Resource and Utilization, Ministry of Agriculture, South China Agricultural University, Guangzhou, 510642, China.

^c^ Institute of Communication and Global Public Opinion, Xi’an International Studies University, Xi’an, 710061, China.

^d^ State Key Laboratory of Urban and Regional Ecology, Research Center of Eco-Environmental Science, Chinese Academy of Sciences, Beijing, 100085, China.

^e^ School of Management, Curtin University, Australia.

*Corresponding authors

Jun Li [lijun267@sysu.edu.cn](mailto:lijun267@sysu.edu.cn) ; Zhuo Chen jintak1988@hotmail.com

^#^JL and FYX contributed equally to the manuscript.

**A1**

Life cycle inventory of three crops cultivation systems. Values are presented as per functional unit.

|  | Categories | unit | Maize | Sorghum | Hybrid Pennisetum |
| --- | --- | --- | --- | --- | --- |
|  | Seeds | kg | 7.92 | 0.67 | - |
|  | Fertilizer ^a^ | kg | 55.44 | 19.71 | 97 |
| **Input** | Pesticide | kg | 1.32 | 0.05 | 2.03 |
|  | Diesel ^b^ | kg | 36.74 | 12.65 | 21 |
|  | Electricity ^c^ | kwh | 680.62 | 141.6 | 1462.9 |
|  | yield | ton | 13.3 | 4.05 | 14.98 |
| **Emission to air** | |  |  |  |  |
|  | Carbon monoxide | g | 23.25 | 7.99 | 13 |
|  | Nitrogen oxides | kg | 1.89 | 0.73 | 2.379 |
| **Output** | Sulfur oxides | g | 147.74 | 50.62 | 84 |
|  | Carbon dioxide | kg | 117.78 | 40.29 | 67 |
|  | Methane | g | 0.25 | 0.09 | 0.15 |
|  | N_2_O | kg | 1.57 | 0.62 | 2.11 |
|  | NMVOC | g | 3.70 | 1.27 | 2.1 |
|  | Ammonia | g | 180.34 | 71.26 | 241 |
| **Emission to water** | |  |  |  |  |
|  | Nitrate | kg | 2.10 | 0.82 | 2.795 |
|  | Phosphorus, total | g | 107.57 | 37.96 | 330 |

^a^ N/P_2_O_5_/K_2_O kg

^b^ machinery utilization including harrow, harvest, irrigation

^c^ machinery utilization for irrigation

Data Source: ([Huo et al., 2016](#_ENREF_5)); ([Gao et al., 2012](#_ENREF_3));([Mochizuki et al., 2014](#_ENREF_12));([Brentrup et al., 2004](#_ENREF_1));([Gaynor and Findlay, 1995](#_ENREF_4)); ([Xiao et al., 2004](#_ENREF_15)); ([Liang et al., 2009](#_ENREF_8))

**A2**

Life cycle inventory of anaerobic digestion. Values are presented as per functional unit.

|  | Categories | unit | maize | sorghum | Hybrid Pennisetum |
| --- | --- | --- | --- | --- | --- |
|  | Lubricant | g | 277.7 | 245 | 277.2 |
|  | Heat | MJ | 1569.38 | 478.15 | 1765.88 |
| **Input** | Diesel | kg | 5.36 | 1.63 | 6.03 |
|  | Electricity | kwh | 61.7 | 18.8 | 69.42 |
|  | biogas | m^3^ | 526 | 556 | 500 |
| **Emission to air** | |  |  |  |  |
|  | Carbon monoxide | g | 3.37 | 1.03 | 3.79 |
|  | Nitrogen oxides | g | 25.7 | 7.83 | 28.92 |
| **Output** | Sulfur oxides | g | 21.42 | 6.53 | 24.10 |
|  | Carbon dioxide | kg | 17.08 | 5.2 | 19.22 |
|  | Methane | kg | 13.57 | 11.95 | 13.55 |
|  | Nitrous Oxide | g | 315.69 | 278.03 | 278.1 |
|  | NMVOC | g | 0.54 | 0.16 | 0.6 |
|  | Ammonia | kg | 42.92 | 37.81 | 42.84 |

**Data Source**: ([Jury et al., 2010](#_ENREF_6)); ([Meyer-Aurich et al., 2012](#_ENREF_11)); ([Lukehurst et al., 2010](#_ENREF_10)); ([Yi et al., 2015](#_ENREF_17))

**A3**

Life cycle inventory of digestate. Values are presented as per functional unit

|  | Categories | unit | maize | sorghum | Hybrid Pennisetum |
| --- | --- | --- | --- | --- | --- |
|  | Phosphate Fertilizer | kg | 199.04 | 270.5 | 517 |
|  | Nitrogen Fertilizer | kg | 486.3 | 661 | 1263 |
|  | Potash Fertilizer | kg | 170 | 231 | 441 |
| `Emission to air | |  |  |  |  |
|  | Methane | mg | 828 | 1125 | 2150 |
|  | Ammonia | g | 6531 | 8875 | 16960 |
| Output | Nitrous Oxide | g | 510 | 692 | 1323 |
|  | Nitrogen oxides | g | 589 | 800 | 1529 |
|  | Carbon monoxide | g | 77 | 105 | 200 |
|  | NMVOC | mg | 12325 | 16750 | 32008 |
|  | Sulfur oxides | mg | 49117 | 66750 | 127556 |
|  | Carbon dioxide | kg | 395.5 | 537.5 | 1027 |
| Emission to water | |  |  |  |  |
|  | Nitrate compounds | g | 6530 | 8875 | 16960 |
|  | Nickel | g | 653 | 887.5 | 1696 |
|  | Copper | g | 618 | 840 | 1605 |
|  | Cadmium | g | 26 | 35 | 67.6 |
|  | Chromium | g | 65.3 | 88.75 | 170 |
|  | Plumbum | g | 260.3 | 354 | 676 |

**Data Source:**([Lukehurst et al., 2010](#_ENREF_10)); ([Wang et al., 2002](#_ENREF_14)); ([Liu et al., 2003](#_ENREF_9)); ([Zeng et al., 1994](#_ENREF_18)); ([Xu et al., 2012](#_ENREF_16)); ([Su, 2005](#_ENREF_13)); ([Yi et al., 2015](#_ENREF_17))

**A4**

Life cycle inventory emissions

|  | Unit | S1 | S2 | S3 |
| --- | --- | --- | --- | --- |
| NOX | g | 113.4 | 110.16 | 124.2 |
| methane | g | 67.83 | 65.892 | 74.29 |
| NMVOC | g | 2.94 | 2.856 | 3.22 |
| Carbon monoxide | g | 57.33 | 55.692 | 62.79 |
| Nitrous oxide | g | 0.8316 | 0.80784 | 0.9108 |
| Formaldehyde | g | 4.4415 | 4.3146 | 4.8645 |
| sulfur dioxide | g | 3.99 | 3.876 | 4.37 |

**Data source**：([Kristensen et al., 2004](#_ENREF_7))；([Eggleston et al., 2006](#_ENREF_2))

**A5**

Weather data for water footprint calculation

| City：Yantai Altitude：20m Latitude：37^。^N Longitud:120^。^E | | | | | | | | |
| --- | --- | --- | --- | --- | --- | --- | --- | --- |
|  | Min Temperature | Max Temperature | Humidity | wind | Sun | Rainfall | Rainfall |  |
|  | ℃ | ℃ | % | km/day | hours | mm | Days |  |
| January | -12.8 | 14.6 | 68 | 363 | 6.0 | 11.5 | 6.4 |  |
| February | -12.6 | 19.8 | 66 | 346 | 6.4 | 9.7 | 5 |  |
| March | -8.1 | 25.0 | 41 | 337 | 7.1 | 16.5 | 4.7 |  |
| April | -2.6 | 31.9 | 43 | 363 | 7.4 | 35.7 | 6.2 |  |
| May | 6.6 | 35.3 | 56 | 346 | 7.9 | 49.9 | 6.2 |  |
| June | 11.5 | 38.0 | 61 | 294 | 7.3 | 70.0 | 8.5 |  |
| July | 14.7 | 36.9 | 77 | 251 | 5.9 | 150.0 | 11.7 |  |
| August | 15.0 | 36.2 | 65 | 251 | 7.2 | 161.6 | 10.3 |  |
| September | 10.7 | 32.4 | 60 | 277 | 7.3 | 83.7 | 7.1 |  |
| October | 0.8 | 30.4 | 49 | 337 | 7.1 | 39.0 | 6.2 |  |
| November | -4.9 | 25.1 | 49 | 372 | 6.3 | 25.1 | 6.2 |  |
| December | -10.8 | 18.8 | 46 | 380 | 5.9 | 6.2 | 7.5 |  |
| Average | 0.6 | 28.7 | 57 | 326 | 6.8 | 55.1 | 7.2 |  |

**A6**

**List of Acronyms and Abbreviations**

AD Anaerobic digestion

ALO Agricultural land occupation

CC Climate change

CHP Combined heat and power

DM Dry materials

FAO Food and Agriculture Organization

FD Fossil depletion

FE Freshwater eutrophication

FET Freshwater ecotoxicity

FU Functional unit

GHG Greenhouse gas

GWP Global warming potential

HT Human toxicity

IPCC Intergovernmental Panel on Climate Change

IR Ionizing radiation

LCA Life cycle assessment

MD Metal depletion

ME Marine eutrophication

MET Marine ecotoxicity

NLT Natural land transformation

OD Ozone depletion

PMF Particulate matter formation

POF Photochemical oxidant formation

TA Terrestrial acidification

TET Terrestrial ecotoxicity

TS Total solid

ULO Urban land occupation

VS Volatile solid

WD Water depletion

WF Water footprint

WFb Blue water footprint

WFg Green water footprint

WFgr Grey water footprint

**References**

1. Brentrup, F., Küsters, J., Lammel, J., Barraclough, P., Kuhlmann, H., 2004. Environmental impact assessment of agricultural production systems using the life cycle assessment (LCA) methodology II. The application to N fertilizer use in winter wheat production systems. European Journal of Agronomy 20(3), 265-279.
2. Eggleston, S., Buendia, L., Miwa, K., Ngara, T., Tanabe, K., 2006. 2006 IPCC guidelines for national greenhouse gas inventories. Institute for Global Environmental Strategies Hayama, Japan.
3. Gao, H., Hu, S., Li, Y., Chen, D., Zhu, B., 2012. Greenhouse gas emission of sweet sorghum ethanol in life-cycle. Transactions of the Chinese Society of Agricultural Engineering 28(1), 178-183.
4. Gaynor, J., Findlay, W., 1995. Soil and phosphorus loss from conservation and conventional tillage in corn production. Journal of environmental quality 24(4), 734-741.
5. Huo, L., Zhao, L., Meng, H., Yao, Z., Cong, H., Wang, G., 2016. life cycle assessmnet analysis for cogeration of fuel gas and biochar. Transactions of the Chinese Society of Agricultural Engineering 26(1), 261-266.
6. Jury, C., Benetto, E., Koster, D., Schmitt, B., Welfring, J., 2010. Life Cycle Assessment of biogas production by monofermentation of energy crops and injection into the natural gas grid. Biomass and Bioenergy 34(1), 54-66.
7. Kristensen, P.G., Jensen, J.K., Nielsen, M., Illerup, J.B., 2004. Emission factors for gas fired CHP units< 25 MW. IGRC, November.
8. Liang, L., Chen, Y., Gao, W., Sui, P., Chen, D., Zhang, W., 2009. Life cycel assessment impact assessment in winter wheat-summer maize system in north China plain. Journal of Agro-Environemnt Science 28(8), 1773-1776.
9. Liu, X., Ju, X., Zhang, F., Pan, J., Christie, P., 2003. Nitrogen dynamics and budgets in a winter wheat–maize cropping system in the North China Plain. Field Crops Research 83(2), 111-124.
10. Lukehurst, C.T., Frost, P., Al Seadi, T., 2010. Utilisation of digestate from biogas plants as biofertiliser. IEA bioenergy 2010, 1-36.
11. Meyer-Aurich, A., Schattauer, A., Hellebrand, H.J., Klauss, H., Plöchl, M., Berg, W., 2012. Impact of uncertainties on greenhouse gas mitigation potential of biogas production from agricultural resources. Renewable Energy 37(1), 277-284.
12. Mochizuki, J., Yanagida, J.F., Kumar, D., Takara, D., Murthy, G.S., 2014. Life cycle assessment of ethanol production from tropical banagrass (Pennisetum purpureum) using green and dry processing technologies in Hawaii. Journal of Renewable and Sustainable Energy 6(4), 043128.
13. Su, J., 2005. Life cycle assessment on biomass-based ethanol fuel in China. Shanghai Jiaotong University, Shanghai.
14. Wang, C., Liu, X., Ju, X., Zhang, F., 2002. Determination of Ammonia Volatilization from Wheat-mazie rotation system field in North China. Acta Ecologica Sinica 22(3), 359-365.
15. Xiao, X., Feng, Q., Liu, Z., Wang, H., 2004. Enrichment characteristics of heavy metals(Cu、Pb、Zn、Cd) in wheat. Energy Environmental production 18(3), 28-31.
16. Xu, Y., Tian, X., Li, D., 2012. The nutrient content compare of different raw biogas fermentation residues. Bulletion of Agricultural Science and Technology 5, 100-102.
17. Yi, R., Zhang, W., Zhou, J., Li, W., Wu, S., 2015. Environmental impact analysis on the production and utilization of digestate based on LCA method. Renewable Energy Resources 33(2), 301-307.
18. Zeng, J., Wang, Z., Zhang, Y., 1994. Flux of N2O emission from the fields in a wheat and maize rotation system. Chinese Journal of Environmental Science 16(1), 32-67.
